# Assessment of the antiproliferative and apoptotic roles of sulfonamide carbonic anhydrase IX inhibitors in HeLa cancer cell line

**DOI:** 10.1080/14756366.2018.1524380

**Published:** 2018-10-26

**Authors:** Ismail Koyuncu, Ataman Gonel, Mustafa Durgun, Abdurrahim Kocyigit, Ozgur Yuksekdag, Claudiu T. Supuran

**Affiliations:** a Department of Biochemistry, Faculty of Medicine, Harran University, Sanliurfa, Turkey;; b Department of Chemistry, Faculty of Arts and Sciences, Harran University, Sanliurfa, Turkey;; c Department of Medical Biochemistry, Faculty of Medicine, Bezmialem Vakif University, Istanbul, Turkey;; d Neurofarba Dept., Section of Pharmaceutical and Nutriceutical Sciences, Università degli Studi di Firenze, Sesto Fiorentino (Florence), Italy

**Keywords:** Carbonic anhydrase IX, sulfonamide, apoptosis, HeLa cell, anticancer agent

## Abstract

Carbonic anhydrase IX (CA IX) has recently been validated as an antitumor/antimetastatic drug target. In this study, we examined the underlying molecular mechanisms and the anticancer activity of sulfonamide CA IX inhibitors against cervical cancer cell lines. The effects of several sulfonamides on HeLa, MDA-MB-231, HT-29 cancer cell lines, and normal cell lines (HEK-293, PNT-1A) viability were determined. The compounds showed high cytotoxic and apoptotic activities, mainly against HeLa cells overexpressing CA IX. We were also examined for intracellular reactive oxygen species (ROS) production; intra-/extracellular pH changes, for inhibition of cell proliferation, cellular mitochondrial membrane potential change and for the detection of caspase 3, 8, 9, and CA IX protein levels. Of the investigated sulfonamides, one compound was found to possess high cytotoxic and anti-proliferative effects in HeLa cells. The cytotoxic effect occurred via apoptosis, being accompanied by a return of pHe/pHi towards normal values as for other CA IX inhibitors investigated earlier.

## 1. Introduction

Significant side effects, therapeutic inefficacy, and partial selectivity are important limitations of current chemotherapeutic agents used for the treatment of cancer^1^^,^^2^. Such shortcomings represent the driving force for the increasing pursuit to search novel therapeutic alternatives for this disease. Investigations looking into effective and tumour-selective chemotherapeutic agents represent an important part of cancer research. Recent studies have suggested that carbonic anhydrase IX (CA IX) enzyme may have such attributes, and the use of the inhibitors of this enzyme has provided promising results for the diagnosis and treatment of cancer^1–5^. Thus, CA IX may be considered as a novel and clinically appropriate therapeutic target with at least one small molecule CA IX inhibitor, SLC-0111, in Phase Ib/II clinical trials for the treatment of metastatic, solid tumors^1^^,^^2^. CA IX represents an important component of the tumour pH regulation machinery that is induced by hypoxia. Its regulatory activities result in the development of a chemical buffer system which involves the hydration of CO_2_ into H^+^ and HCO_3_^−^. This buffer system is actively transported into the cell, assisting in the maintenance of intracellular pH (pHi) as well as contributing to the extracellular acidosis (pHe) characteristic of many solid tumors^1–5^. Therefore, while cancer cells are protected from the damage caused by the glycolytic mechanisms, they can also exhibit metastatic activity owing to the extracellular acidity. Thus, an intervention on one or more effectors of this system is thought to have potent inhibitory effects on tumour growth^1^^,^^2^^,^^6–8^.

CA IX inhibition by many classes of inhibitors (e.g. sulfonamides, coumarins) has been shown to halt the proliferation of cancer cells *in vitro* and to inhibit metastasis without nonspecific toxicity in several tumour models^1–5^. Furthermore, combination of such inhibitors with conventional chemotherapy or radiotherapy has also been demonstrated to inhibit the growth of several tumors^2^^,^^9–12^. Sulfonamides exhibit a wide array of biological activities, with recent *in vitro* and *in vivo* demonstration of anti-cancer activity. Anti-cancer activity occurs via a number of mechanisms, the most important of which is the inhibition of tumour-associated CA isoforms, such as CA IX and XII^1^^,^^11^.

In a previous study, we have reported the synthesis and inhibitory activity against carbonic anhydrase isoforms I, II, IX, and XII of new sulfonamide derivatives^13^. Furthermore, their cytotoxic effects were examined on several cancer cell lines as well as normal cells^14^^,^^15^. In this study, six different synthesised imine and amine sulfonamide derivatives with documented CA IX inhibitor activity^13^ have been tested in terms of their cytotoxic effects in cancer cells (HT-29, HeLa and MDA-MB-231), and in normal cells (PNT1A, HEK-293). The underlying molecular mechanisms of the potential anti-tumoral activity of the CA IX inhibitor sulfonamide **A1** with strong cytotoxic effects were also assessed, including the cellular proliferation, intracellular radical and mitochondrial membrane potential, intra-/extracellular pH changes, apoptosis, and autophagy.

## 2. Materials and methods

The cell culture medium (RPMI 1640), DMEM-F12, foetal bovine serum (FBS), streptomycin, and penicillin, were purchased from Gibco BRL (Life Technologies, Paisley, Scotland); WST-1 (Roche, Germany), ROS kit (Abcam, Cambridge, UK), MPP kit, ethidium bromide, acridine orange, trypsin– EDTA solution, and dimethyl sulfoxide (DMSO), from Sigma Chemical Company (Germany) and the culture plates from Nunc (Brand products, Denmark).

### 2.1. Cell culture and drug preparation

Cancer and normal cell lines were purchased from ATCC and stored in liquid nitrogen. HT-29 (colon adenoma cancer), HeLa (cervix adenoma cancer cell), MDA-MB-231 (breast adenoma cancer cell) and HEK-293 (embryonic kidney epithelial cell), PNT-1A (normal prostate cells) cell lines were incubated in DMEM: F-12 and RPMI-1640, including 10% Foetal Bovine Serum (FBS), 100 μg/mL streptomycin/100 IU/mL penicillin, at 37 °C in an incubator containing 5% CO_2_, 95% air in a humid atmosphere.

The aromatic sulfonamides used in this study were reported in our previous study^13^. Briefly, the imine compound derivatives (**A1-A3**) were synthesised through the reaction of 4–(2-aminoethyl)benzenesulfonamide with substituted aromatic aldehydes with catalytic amounts of formic acid in methanol at the refluxing temperature for 3–5 h. The secondary amine derivatives (**B1-B3**) were prepared by reduction of the imine compounds (**A1-A3**) with NaBH_4_ in methanol. All the derivatives of imine and amine were characterised with both analytical and spectral data. The aromatic aldehydes used in the synthesis were 5-chloro-2-hydroxybenzaldehyde **(A1,B1)**, 3,5-dichloro-2-hydroxybenzaldehyde **(A2, B2)**, and 2-hydroxybenzaldehyde **(A3, B3)**. These CA inhibitors have been shown to induce a moderately effective, reversible inhibition of the membrane-bound isozyme CA IX compared with traditional inhibitors. The K_I_s of the CA inhibitors and the chemical structures of the inhibitors tested are shown in Table 1^13^.

**Table 1.** Structures and K_i_ values against four CA isoforms of sulfonamide compounds **A** and **B**^13^.

							

### 2.2. Cytotoxicity analysis

The cytotoxic effects of the substances were evaluated with WST-1 kits (Roche, Germany) in accordance with the manufacturer’s protocols. The cells were plated on 96-well plates (10^4^ cells in each well). After incubation for 24 h, the media were discarded and the substances and Cisplatin as the control drug, at doses of 0, 2.5, 5, 10, 25, 50, 100, and 200 μM, were incubated for 24, 48, and 72 h. WST-1 reactive of 10 µl was added to all wells. Following 4 h incubation, the measurements were taken on a plate reader (Spectramax M5) at wavelengths of 450 and 630 nm. Graphs were then created and the IC_50_ value of each substance was calculated.

### 2.3. Investigation of antiproliferative effects

The effects of the substances on the proliferation of HeLa cells were investigated using a commercial proliferation kit 5-bromo-2′-deoxyuridine (BrDU) (BioVision, Wehrheim, Germany)) according to the manufacturer’s protocols. After 24 h, the cells were plated on 96-well plates (10^4^ cell/ml), the medium was replaced and the substances were administered at doses ranging from 2.5 to 200 μM. After 72 h, the media were discarded, 100 µl 1X of BrdU reactive was added, and the samples were incubated in an incubator, containing 5% CO_2_, 95% fresh air, at 37 °C for 4 h. The medium was then removed, and 100 μl of fixative/denaturation solution was added and incubated for 30 min. 100 µl 1X of the antibody solution BrdU was added, and following 1 h incubation, was washed twice with 300 µl 1X of the washing solution. 100 μl 1X of anti-mouse HRP-linked antibody was added and incubated at room temperature for 1 h. Then the cells were washed three times with 300 µl 1X of washing solution. The final incubation was performed with 100 μl of TMB substrate solution for 5 min, after which the stop solution was added, and then the reading was taken at a wavelength of 450 nm.

### 2.4. Apoptosis detection by annexin V-FITC

The apoptotic effect of the substances was explored with the commercial FITC Annexin V Apoptosis Detection Kit I (BD Biosciences, New Jersey, USA) according to the manufacturer’s protocol. The cells were plated on 6-well plates (5 × 10^5^ cells/well), and following 24 h incubation, sulfonamide compounds at concentrations of 10 and 25 μM were administered, followed by incubation for 24 h. The cells were raised by trypsin handling, and transferred into new tubes within 1X binding buffer, as 1 × 10^6^ in each tube, which was incubated for 15 min at room temperature. 5 μL of fluorochrome-conjugated Annexin V and 5 μL of Propidium Iodide dyes were then added. 100 μl 1X binding buffer was added to the cells, and they were then centrifuged at 1200 rpm, for 5 min. Finally, the cells were analysed in flow cytometry (BD Via, New Jersey, USA).

### 2.5. Detection of intracellular ROS production

Intracellular free radicals were measured using the Cellular Reactive Oxygen Species Detection Assay commercial kit (Red Fluorescence, Abcam, Cambridge-UK, 186027). In this kit, a fluorescence probe that is permeable to the cell is used. When the probe reacts with ROS, it produces red fluorescence. The process was applied following kit protocol. The cells were plated on black plates, as 3 × 10^4^ in 100 µl, and incubated for 24 h. Sulfonamide compounds in PBS were administered at doses of 0–25 μM and incubated at 37 °C in an atmosphere of 5% CO_2_ for 1 h (protected from light). After the compounds were poured out, the wells were filled with 100 µl of ROS Deep Red Working Solution and incubated at 37 °C in an atmosphere of 5% CO_2_ for 1 h. A fluorometry device (Spectramax, M5) Ex/Em = 650/675 nm (cut off = 665 nm) was used to take the measurements.

### 2.6. Detection of mitochondrial membrane potential (MMP)

Mitochondrial membrane potential (MMP) changes appearing in the intrinsic pathway of mitochondria were spectrophotometrically detected. MMP was measured in a Spectramax, M5 fluorimetric device, at wavelengths of 490 nm (excitation), 530 nm(emission) according to the fluorometric Mitochondria Staining Kit (Sigma-Aldrich, Schnelldorf, Germany, CS0390) protocol.

### 2.7. Detection of intracellular pHi, extracellular pHe and lactate level

Intracellular pH was measured according to the protocol of the fluorometric Intracellular pH Assay Kit (Sigma, Schnelldorf, Germany, MAK-150). Fluorescent BCFL-AM indicator that was able to pass through the cell membrane was used to measure intracellular pH fluctuations in the fluorometric intracellular pH kit. BCFL-AM was utilised to measure the reductions in intracellular pH of the cells that had been treated by various conditions. The cells were plated on black plates, as 6 × 10^4^ in each well of the plate, and incubated for 24 h. The medium was replaced with BCFL-AM Reagent which had been prepared in 100 µl of HBS solution (Hank’s buffer with 20 mM HEPES, 5 mM Probenecid), then the cells were incubated at 37 °C in an atmosphere of 5% CO_2_ for 30 min (protected from light). Sulfonamide compound at doses of 0–25 μM was added to the HBS solution. In the next 5 min, the measurement was performed at wavelengths of 490 nm (excitation) 535 nm (emission) in spectrofluorimetry (Spectramax, M5). Extracellular pHe and lactate levels were measured with commercial kit by the blood gas device (ABL90 FLEX PLUS, Radiometer, Copenhagen, Denmark) which is routinely used in the clinical biochemistry laboratory of hospital. After the application of the substance in this method, the media were analysed directly by the device.

### 2.8. Acridine orange and ethidium bromide (AO/EB) staining assay

At 24 h after the plating of HeLa cells in 12-well plates at 5 × 10^4^ cell/ml, sulfonamide derivative compounds at concentrations of 10 and 25 μM were added to the plates, and the cells were incubated at 37 °C for 24 h. The cells were then washed with PBS, and released for incubation in a solution including 100 μl (acridine orange (100 µg/ml) and ethidium bromide (100 µg/ml) at room temperature for 5 min. The morphological fluctuations indicating apoptosis were investigated under fluorescent microscopy (Olympus CKX 53, DP73, Tokyo, Japan).

### 2.9. Detection DNA damage (*γ*-H2AX) by immunofluorescence staining

HeLa cells (1 × 10^4^/ml) were seeded in 12-well cell culture dishes and after 24 h they were administered with the sulfonamide derivative compounds at doses of 10 and 25 µM and incubated for 24 h. The cells were washed with PBS and fixed with 100% methanol at −20 °C overnight, which was followed by PBS washing three times for 10 min for each washing. The cells were treated with 0.2% Triton X-100 in a shaker, at room temperature for 5 min. They were washed with PBS, and 1% BSA-containing PBS was then added, and incubation with primary mouse monoclonal anti-*γ*-H2AX antibody (Cell Signalling Technology, Danvers, MA, USA) was applied at 37 °C for 1 h. The cells were washed with PBS for 5 min three times. Secondary goat anti-mouse Alexa-488-conjugated IgG (Invitrogen, Thermo Fisher Scientific, Waltham, MA, USA) was administered and the cells were incubated at 37 °C for 20 min, followed by PBS washing for 4–5 min three times. 1 ml 70% EtOH was added, and at +4 °C, released to incubation for 5 min. 1 ml 100% EtOH was added in a shaker, which was then incubated at room temperature for 1–2 min. The nucleus was stained with DAPI and the images were recorded using an Olympus Inverted fluorescence microscope (Olympus CKX 53, DP73, Tokyo, Japan).

### 2.10. Western blot analysis

The cells were seeded on 6 cm^2^ cell culture dishes, and treated with sulfonamide compounds, at doses of 10 and 25 μM, for 24 h. The cells were then washed with cold PBS, which was followed by lysis with RIPA lysis buffer (10 mM Tris-HC1 pH =8, 1 mM EDTA, 1 mM EGTA, 140 mM NaCl, 1% TritonX-100, 0.1 SDS, 0.1% Sodium deoxycholate), 1X phosphates and protease inhibitor (Santa Cruz Biotechnology, Dallas, USA). The acquired supernatants from the lysates which were centrifuged at 12000 g, +4 °C, for 10 min, were transferred to new tubes. Protein concentration was detected using the protein assay kit BCA (Thermo Fisher Scientific, Waltham, MA). After a 30-min period of protein (50 µg) standing in 10% SDS-PAGE gel at 50 V and at 80 V for 3 h, those were blotted to PVDF membrane at 70 V for 5 min (Bio-Rad Turbo Transfer System, Segrate (MI), Italy). After embedding in 1X TBST (12,1 gr Tris, pH 7.5, 70 gr NaCl, 0.1% Tween-20) containing 5% dry milk or 5% BSA, the primer monoclonal antibodies Cleaved Caspase 3,8,9, Cleaved PARP (Millipore) CA IX and Beta-actin, were applied to the membrane and left overnight, which was followed by washing with 1X TBS-T and an incubation period with secondary antibodies of HRP rabbit (Cell signaling, Danvers, MA, USA) or mouse (1/10.000) for 60 min. The membrane was again washed with 1X TBS-T. For band imaging, ECL substrate (EMD Millipore Corp., Billerica, MA, USA) was added and observations were made via the imaging system (LI-COR Odyssey Fc, Lincoln, NE, USA).

### 2.11. Measurement of intracellular free amino acids by LC-MS/MS

The intracellular free amino acid level was measured according to the Chen et al method^16^. HeLa cells were planted in 10 cm^2^ cell culture dishes, and following 24 h treatment with sulfonamide at a dose of 25 μM, the growth medium was poured out, and the cells were swiftly washed twice with 5 ml cold PBS. Pre-cooled MeOH (−60 °C) was added, and the cells were stripped with a cell scraper. The cell suspension obtained was replaced in 15 ml conic tubes. The samples were kept in liquid nitrogen for 10 min and then dissolved on ice, which was triplicated until the cells were shredded. The samples were centrifuged at 3.000 g at 4 °C, for 30 min, and the supernatants were transferred to new tubes. The amino acid level in the supernatant was measured using LC-MS/MS according to the protocol of the Jasem kit. The Jasem free amino acid assay kit is used for studies involving the diagnosis of various hereditary metabolic disorders and the feeding of newborns with hereditary metabolic disorders. In this study, the protocol used to determine the intracellular free amino acid is as follows. In a new tube, 50 µl supernatant, 50 µl internal standard solutions, and 700 µl Reagent-1 were mixed by vortex for 10 s, and the acquired solution was centrifuged at 4000 rpm for 5 min. 27 amino acids in the acquired supernatant were analysed in HPLC vials using LC-MS/MS (Shimadzu 8045, Kyoto, Japan). The residual pellet was lysed in 1 ml lysis buffer, protein concentration of which was detected using the BCA protein assay kit (Thermo Fisher Scientific, Waltham, MA, USA). Finally, the total protein levels were normalised and the net amino acid levels in the supernatants were defined.

### 2.12. Real-time quantitative PCR

All gene expression levels of the cells were examined following 24 h incubation with sulfonamide compound **A1** at a dose of 25 μM. Total RNAs were isolated using the miRNeasy mini kit (Qiagen, Hilden, Germany) and reverse transcription was performed with the Ipsogen RT Set (Qiagen, Hilden, Germany) according to the kit protocol. RT-qPCR was then performed using the QuantiTect SYBR Green PCR kit (Qiagen, Hilden, Germany) in the Rotor-Gene Q real-time PCR system (Qiagen, Hilden, Germany). Each sample was studied in triplicate, using primer sets **Caspase-3** (F: GAGCACTGGAATGTCATCTCGCTCTG and R: TACAGGAAGTCAGCCTCCACCGGTATC), **Caspase-8** (F: CATCCAGTCACTTTGCCAGA and R:GCATCTGTTTCCCCATGTTT), **Caspase- 9** (F:ATTCCTTTCCAGGCTCCATC and R: CACTCACCTTGTCCCTCCAG), **caspase-12** (F:GCCATGGCTGATGAGAAACCA and R: TCGCATCCCCAAAAGGTCAA), **CA IX** (F:AGTCATTGGCGCTATGGAGG and R: TCTGAGCCTTCCTCAGCGAT), **NRF-2** (F: TTCGGCTACGTTTCAGTCAC and R: TCACTGTCAACTGGTTGGGG), **BAX** (F: TCCATTCAGGTTCTCTTGACC and R: GCCAAACATCCAAACACAGA), **BCL-2** (F: ATCGTCGCCTTCTTCGAGTT and R: ATCGTCGCCTTCTTCGAGTT), **LC-3** (F: ATCATCGAGCGCTACAAGGG and R: AGAAGCCGAAGGTTTCCTGG), **BECLİN-1** (F: CGACTGGAGCAGGAAGAAG and R: TCTGAGCATAACGCATCTGG), and **GAPDH** (F: GGAAGGACTCATGACCACAGT and R: GGATGATGTTCTGGAGAGCCC). Gene expressions were calculated with the 2-ΔΔCt method and were compared to control groups. The GraphPad Prism 6 programme defined the *p*-values. Primers were designed using Primer blast on the National Centre for Biotechnology Information website (https://blast.ncbi.nlm.nih.gov/Blast.cgi). All primers were determined to be 95–100% efficient and all exhibited only one dissociation peak.

### 2.13. Cell cycle assay

Cell cycle analysis was performed using BD Cycletest^™^ Plus DNA Reagent kit (BD Biosciences, New Jersey, USA). According to the kit protocol, 1 × 10^6^ cells were plated on 6-well plates, and following 24 h, the cells were incubated for 24 h, with sulfonamide derivative compounds at concentrations of 10–25–50 μM. The cells were raised with trypsin handling and centrifuged at 1500 rpm for 5 min. A suspension was formed with 1X binding buffer, and respectively, 250 µl solution A was added and incubated in a light-free environment for 10 min; 200 µl solution B was added and incubated in a light-free environment for 10 min; and 200 µl solution C was added and incubated in at +4 °C in a protected-light environment for 10 min. All analyses were performed by flow cytometry (BD Via, New Jersey, USA).

## 3. Results

### 3.1. The effects of the investigational compounds on cell viability and proliferation

In this study, six sulfonamide derivatives, having documented CA IX inhibitor activity, reported by Durgun et al^13^ have been tested in terms of their cytotoxic effects in cancer cells having high (HT-29, HELA), low or no (MDA-MB-231) CA IX production as well as in normal cells (PNT1A, HEK-293), using the WST-1 methodology in time- and dose-dependent manner. The IC_50_ values for these compounds and for Cisplatin as positive control are shown in Table 2.

**Table 2.** Cytotoxicity of derivatives of sulfonamide on tumour cell lines and normal cell lines.

Compound	IC_50_ (*µ*M)^a^														
	Cancer Cell									Normal Cell					
	HeLa			HT-29			MDA-MB-231			HEK-293			PNT-1A		
	24 h	48 h	72 h	24 h	48 h	72 h	24 h	48 h	72 h	24 h	48 h	72 h	24 h	48 h	72 h
A-1	28.9	17.4	13.7	299.1	52.6	123.1	293.1	65.9	37.3	105.3	209.3	25.3	388.9	110.5	202.6
B-1	217.7	111.9	98.9	606.4	63.8	91.8	492.2	595.3	243.3	323.8	206.9	63.7	593.1	189.7	371.6
A-2	345.4	300.2	123.3	234.3	254.34	134.3	125.4	145.4	100.3	120.3	100.2	65.44	167.4	170.4	100.3
B-2	234.3	212.2	154.3	356.5	200.3	155.4	145.4	43.3	54.3	230.4	215.4	120.3	55.4	23.2	12.3
A-3	123.3	112.3	76.5	143.2	100.3	87.4	98.5	80.45	65.4	98.43	65.44	32.2	45.4	43.3	23.3
B-3	156.5	134.3	45.4	560.4	450.43	210.3	345.4	234.3	126.5	178.6	120.2	45.4	100.3	89.4	70.4
Cisplatin	28.9	7.6	6.1	23.7	20.0	16.3	21.2	10.2	7.0	29.6	14.0	2.0	13.4	11.4	8.2

^a^Values are the means of three independent experiments.

Of the six sulfonamides tested, compound **A1** demonstrated the strongest cytotoxic effects in HeLa cells having high CA IX expression. Sulfonamide **A1** was associated with a dose-dependent inhibition of proliferation of HeLa cells with an IC_50_ value of 13.7 μM, whereas the chemotherapeutic agent cisplatin showed an IC_50_ value of 6.1 μM.

**Figure 1.**
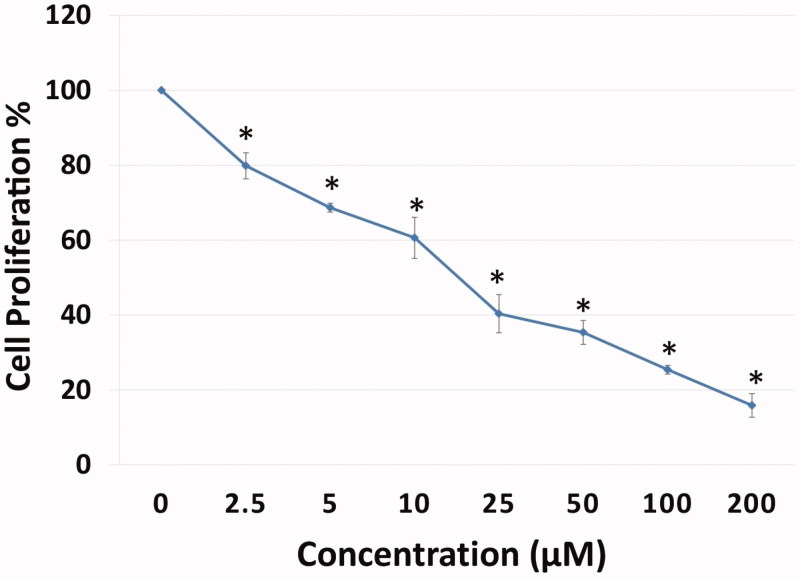
Antiproliferative effect of **A1** against HeLa cell growth *in vitro*: cells were grown in 96-well plates and were treated with various concentrations of compound **A1** for 24 h. All data are expressed as mean ± SD values from three independent experiments. *p*-values of less than *p < .01, compared with the control group) are considered significant.

The cytotoxicity analysis showed that out of the six sulfonamide derivatives, the strongest cytotoxic activity on HeLa cells was achieved by compound **A1** at 72 h (Table 2). Thus, the antiproliferative effect of Compound **A1** with CA IX inhibitory activity on HeLa cells was explored using BrdU ELISA methodology. Compound **A1** was found to have dose-dependent anti-proliferative effects (IC_50_; 13.7 µM) on HeLa cells (Figure 1). 

### 3.2. Determination of apoptotic effects of compound A1 on HeLa with flow cytometry and AO/EB double staining assay

**Figure 2.**
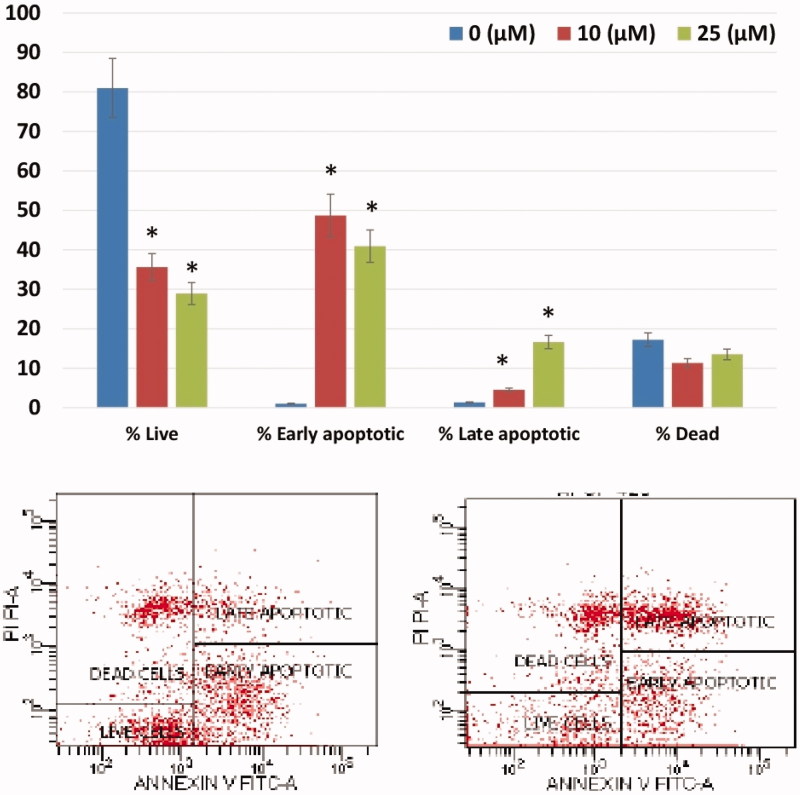
Induction of apoptosis in HeLa cells by sulfonamide **A1**. The apoptosis ratio was analysed by flow cytometry. Data analyses of HeLa cells and contour diagram of Annexin V/PI Flow Cytometry.

The apoptotic effects of the CA IX inhibitor **A1** with cytotoxic effects on HeLa cells were assessed using Annexin-V FITC flow cytometry and Acridine orange/ethidium bromide (AO/EB) methods. The effect of the treatment of HeLa cells with **A1** at different doses (10 and 25 μM) for 24 h was tested using flow cytometric Annexin-V. **A1** was shown to induced apoptosis starting from 10 μM, mostly during the stage of early apoptosis (48.7%) (Figure 2).

AO/EB fluorescence staining is a method frequently used to observe the morphological changes occurring during cell death and the images obtained using this method are shown in Figure 5. While a green cell nucleus denotes viable cells, those with yellowish-orange and fragmented nuclei represent apoptotic cells. The Figure 5 shows the AO/EB results presence of apoptosis in HELA cells treated with **A1** has been confirmed morphologically, corroborating the results of other apoptotic tests.

### 3.3. The effect of A1 on lactate level, intra and extracellular pH balance

CA IX participates in pH regulation in cancer cells, influencing both intra- (pHi) and extracellular pH (pHe)^1–5^. Its overexpression in tumour cells is associated with an increased pHi (up to 7.5) compared to the physiological pH and a decrease of pHe (up to 6.1–6.5), leading to unfavourable effects on cell viability^1–5^^,^^8^^,^^12^. Inhibition of CA IX in cancer cells leads to a normalisation of the pH gradient, with a return of pHe to more normal values^1–5^^,^^17^.

Intracellular pH (pHi) plays an important role in the intracellular hemostasis. Alterations in cellular pHi lead to disruption of mitochondrial MMP and DNA damage associated with increased intracellular ROS, inducing apoptosis^2^^,^^16^^,^^18^. Similarly, since extracellular pH (pHe) has a particularly important role in the metastatic process of cancer cells, an increased pHe may lead to an increased metastatic activity^17^.

Accordingly, the effect of CA IX inhibition induced by **A1** in HeLa cells on lactate level, pHi and pHe were tested using B-CFAM and blood gas measurements. The results are illustrated in Figure 4(A–B). Treatment of HeLa cells with **A1** resulted in a reduction of pHi and a significant increase of the highly acidic initial pHe from 6.4, in a dose-dependent manner (Figure 4(A)), to more normal levels, arriving at a pHe of 7.3. Such changes in pH values are strongly suggestive of an inhibition of CA IX by **A1**.

### 3.4. Assessment of the apoptotic pathway of compound A1 in HELA cells

To test whether apoptosis induced by Compound **A1** on HeLa cells at low doses (10–25 μM) the cleaved caspase 3, caspase 8, caspase 9 method was used; also, in order to determine the CA IX inhibition by cleaved-PARP, the CA IX protein levels were assessed with the Western blot analysis (Figure 7). The obtained results show that **A1**, most notably at a dose of 10 μM, was associated with a significant increase in cleaved- caspase 3, caspase 8, caspase 9, and c-PARP levels. Thus, these results suggest that **A1** induces strong apoptosis in HeLa cells via both intrinsic and extrinsic apoptotic pathways. Although both pathways are triggered, higher levels of c.caspase-9 as compared to c.caspase-8 indicates that the extrinsic pathway may be more actively involved. Also, decreasing mitochondrial membrane potential and BCL-2 and increasing BAX and NRF-2 levels in HeLa cells after administration of **A1** confirm that the intrinsic pathway is more active.

### 3.5. The effect of compound A1 on MMP and ROS alterations

**Figure 3.**
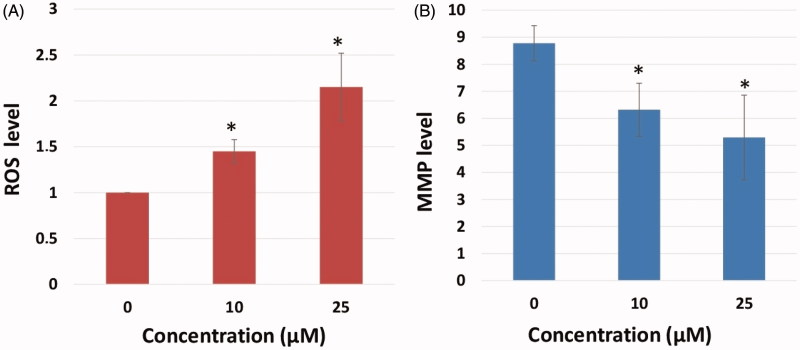
Reactive oxygen species (ROS) levels and mitochondrial membrane potential (MMP) stability were detected by spectrofluorimetry. Cells were treated with various concentrations of compounds (0–25 μM) for 24 h. (A) The cells were stained with the ROS red dye and spectrofluorimetry was performed to determine the intracellular ROS. (B) The cells were stained with the JC-1 dye and spectrofluorimetry was performed to determine the MMP stability. The data are presented as the mean ± SD. (*n* = 3) *p < .01 compared with the control.

Sulfonamide **A1** was found to increase the intracellular ROS in HeLa cells in a dose-dependent manner (Figure 3(A)). Also, the increase in NRF-2 gene, which triggers the intracellular antioxidant system (Figure 9), provides further evidence for the increased intracellular ROS. The loss of mitochondrial membrane potential (MMP) has been particularly associated with the activation of the extrinsic apoptotic cascade^8^^,^^9^. Following treatment of HeLa cells with Compound **A1**, a dose-dependent decrease in MMP was noted (Figure 3(B)).

**Figure 4.**
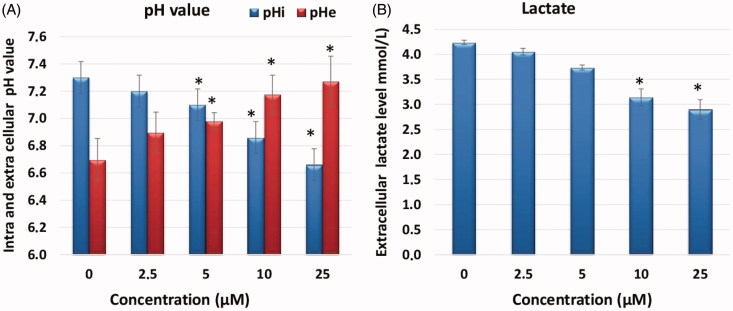
Measurement of intracellular pHi, extracellular pHe and extracellular lactate level. (A) Effects of sulfonamide CA IX inhibitor **A1** on pHi and pHe in HeLa cell and media. (B) Effects of compound **A1** on lactate level in HeLa cell media. The data are presented as the mean ± SD. (*n* = 3) *p < .01 compared with the control.

**Figure 5.**
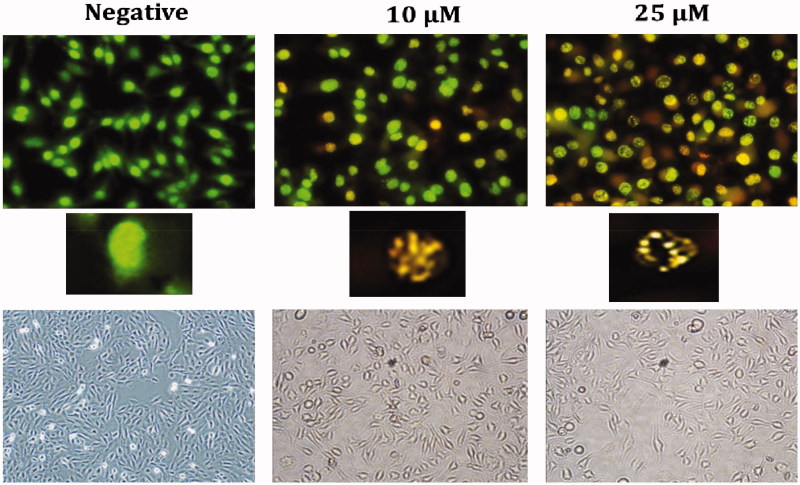
Morphological changes observed under florescence and light microscope in **A1** treated HeLa cells after staining with AO/EB. All data are expressed as mean ± SD values from three independent experiments. *p*-values of less than *p < .05, (compared with the control group) are considered significant.

### 3.6. The effect of compound A1 on the DNA damage of HeLa

**Figure 6.**
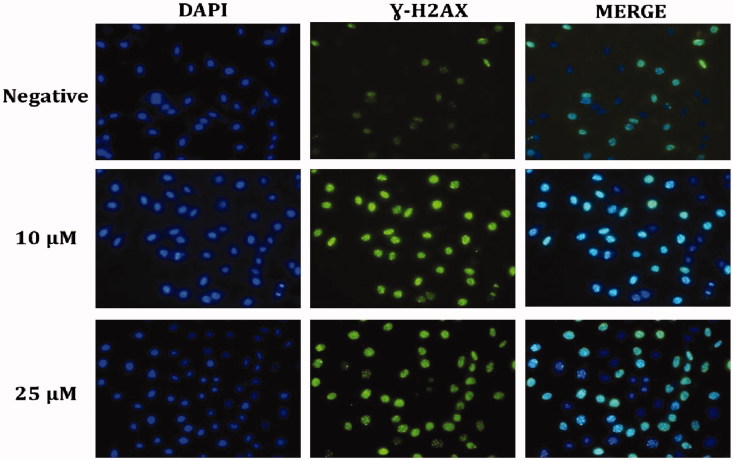
Representative DNA damage fluorescence microscopy images from cell treated with compound **A1**. A fragment of each cell was fixed and processed for *γ*-H2AX immunofluorescent staining. *γ*-H2AX staining is green; nuclei are stained with DAPI blue.

In order to detect DNA damage induced by the augmented ROS after administration of Compound **A1** to HeLa cells, the *γ*-H2AX foci formed in the nuclei were examined using the immunofluorescent method Figure 6. Treatment of HeLa cells with **A1** was found to increase the number of nuclear **γ**-H2AXfoci, also confirming the ROS-induced DNA damage in HeLa cells (Figure 6).

### 3.7. The effect on the cell amino acids metabolism

The effects of CA IX inhibitor **A1** on the amino acids metabolism of HeLa cells was tested by the measurement of free amino acids. After administration of **A1**, LC-MS/MS was used to determine the change in 18 different free amino acids profile in HeLa cells (Figure 8). Upon administration of the inhibitor **A1**, the amino acids showed a decline as compared to the negative control group.

### 3.8. Assessment of the effect of compound A1 on the gene expression of HeLa using real-time PCR

The effect of several proteins, which are markers of apoptosis, autophagy, and free radicals, on gene expression in HeLa cells treated with **A1** (at a dose of 25 μM for 48 h) was examined using the Real-Time PCR method (qRT-PCR), result of which is shown in Figure 9. The increase in the expression genes involved in the apoptotic pathway (Caspase 3, 8, 9, 12, and Bax) after administration of **A1** as well as the decrease in Bcl-2 suggest that the apoptotic mechanisms have been triggered. Furthermore, the increased expression of caspase-8 and caspase-9 genes shows that both internal and external apoptotic pathways were triggered. In addition, a significant increase in NRF-2 gene was noted. Increased NRF-2 gene expression correlates with ROS levels, and Compound **A1** has anti-cancer activities through increased ROS in HeLa cells (Figure 9).

### 3.9. The effect of compound A1 on the HeLa cell cycle

The effect of **A1** on cell cycle is illustrated in Figure 10. Compound **A1** has been found to affect the cell division in HeLa cells at concentrations above 25 μM, with a dose-dependent manner of slowing down the cell division. At a dose of 50 μM, the per cent slowing down in G0/G1, S, and G2/M phases of the cell cycle were of 65.7%, 19.4%, and 13.8%, respectively. As compared to a control, a dose of 50 μM **A1** was associated with halting of cell division in the G0/G1 phase (65.7–56.6) (Figure 10).

## 4. Discussion

Carbonic anhydrase IX (CA IX) is a transmembrane enzyme overexpressed in many different cancer cells that allows a suitable intracellular pH (pHi) for tumour growth and survival in addition to facilitation of the tumour invasiveness via an increased extracellular acidity (pHe). Recent observations showing a high expression of CA IX in many solid tumours as compared to its limited expression in normal tissues have rendered this enzyme a particularly attractive therapeutic target^1–5^^,^^19^. Pharmacological interventions on the catalytic activity of CA IX using a number of sulfonamide derivatives have disrupted the pH balance of cancer cells, leading to a reduction of primary tumour growth and inhibition of metastases formation^1–5^^,^^17^. Thus, a significant proportion of such agents have become a new strategy for cancer treatment, with one such sulfonamide, SLC-0111 in Phase Ib/II clinical trials^20^. In this regard, many studies have been performed to synthesise sulfonamide analogues and derivatives with the purpose of augmentation of the anti-cancer potential and reduction of toxic effects in normal cells^4^^,^^6^^,^^20–24^.

For example, Dubois et al.^22^ found increased anti-tumour activity when CA IX inhibitor sulfonamide and radiotherapy was combined in mice injected with HT-29 colorectal tumour cells. Cianchi et al.^25^ found significant reduction in HeLa and 786-O cell proliferation with induction of apoptosis due to increased ceramide level, using newly synthesised two aromatic sulfonamides, such as TR1 and GA15, in cell lines producing CA IX.

In the current study, the cytotoxic effects six sulfonamide derivatives with CA IX inhibitor properties originally reported by Durgun et al.^13^ on human cancer (HeLa, HT-29 and MDA-MB-231) and normal cell lines (HEK-293, PNT-1A) have been examined in addition to their selective killing potential in CA IX producing HeLa cells.

The results of the cytotoxicity analysis showed that the peak cytotoxic activity on HeLa cells with high CA IX expression was achieved by Compound **A1** among these 7 sulfonamide derivatives. Also, the cytotoxic effects of Compound **A1** on normal cells (PNT-1A) was found to be much lower (IC_50_; 197–398.3 μM) as compared to the cancer cells (HeLa). The selective cytotoxic and anti-proliferative effects of **A1** on HeLa cells suggest that the anti-tumour effects are probably mediated by CA IX inhibition. Similar to our observations, previous studies have also suggested that a variety of CA inhibitors could inhibit the invasiveness of and induce apoptosis in cancer cell lines positive for CA IX^25^^,^^26^.

It has been hypothesised that cancer cells are able to maintain high pHi levels for inhibiting intracellular acidosis, which is a triggering factor for early stage apoptosis^2–4^. Thus, lowered intracellular pH may disrupt the mitochondrial membrane potential (MMP), leading to increased reactive oxygen species (ROS) and DNA damage with consequent cellular apoptosis^6^^,^^8^^,^^16^^,^^18^^,^^27^^,^^28^. In a study by Cianchi et al.^25^ two aromatic sulfonamides (**TR1** and **GA15**) were found to induce apoptosis in cancer cells due to stimulation of oxidative stress by increased ceramide and p38-MAPK, after intracellular acidosis due to CA IX inhibition, in human renal carcinoma cell lines and in HeLa cells, regulating CA IX expression driven by cell density^11^^,^^29^.

Intracellular acidosis in conjunction with increased ROS and NRF-2 as well as MMP loss was observed in the present study in cervical cancer HeLa cells treated with **A1**. Increasing ROS and NRF-2 levels are indicative of increased oxidative stress in these cells. NRF-2 is an important transcription factor regulating the gene sequence of antioxidant enzymes that protects the cells from increased free radicals via activation of the antioxidant systems^14^^,^^15^. In our study, the increase in NRF-2 confirms the increase in intracellular ROS in HeLa cells after administration of sulfonamide **A1**. The function of CA IX with respect to extracellular pH regulation and the disrupted pH balance (pHi and pHe) in HeLa cells treated with **A1** may be considered as evidence for the selective cytotoxic effect of **A1** in HeLa cells occurring as a consequence of CA IX inhibition.

Apoptosis is a natural mechanism for cellular death and represents a promising target for cancer treatment. Both external and internal pathways utilise caspases to execute apoptosis via degradation of hundreds of different proteins. In cancer cells, the apoptotic pathway is typically inhibited by the excessive expression of anti-apoptotic proteins and inadequate production of pro-apoptotic proteins. Most of these alterations are associated with an intrinsic resistance to chemotherapy. The most effective novel anti-cancer agents include those exhibiting anti-cancer activity via the activation of the apoptotic pathway^30^.

**Figure 7.**
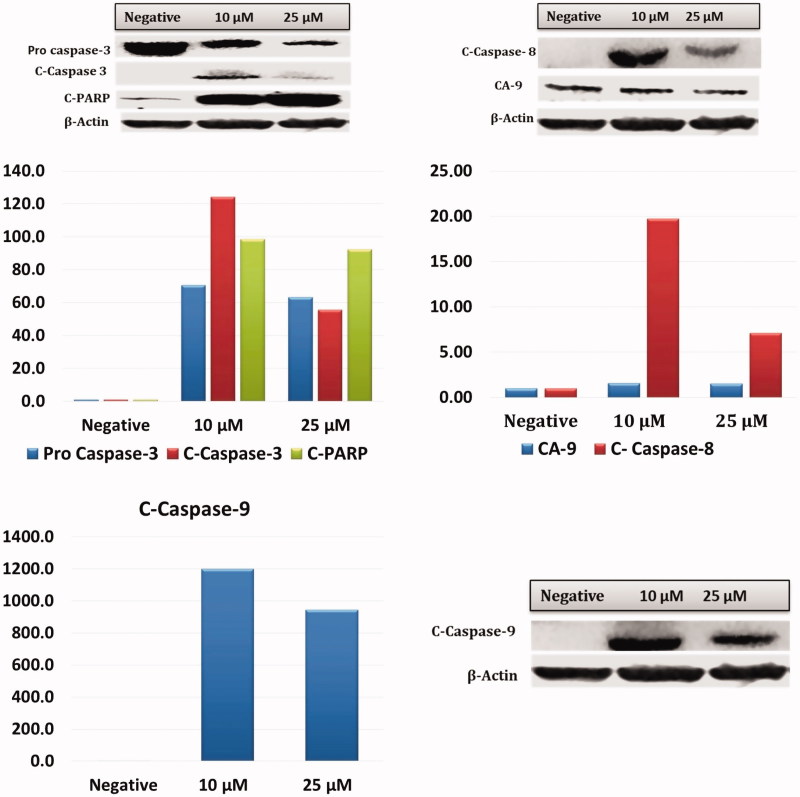
Effect of CA IX inhibitor **A1** on cleaved caspases-3, 8, 9, C.PARP, and CA IX expression levels in HeLa cells. Cells were treated with **A1** (0, 10 and 25 µM) for 24 h. Proteins were normalised to the respective β-actin and is presented relative to the value for the untreated control cells. The densitometry quantification of blot was determined by the software Li-Cor Fc.

The alterations in pH balance in CA IX positive HeLa cells induced by **A1** were found to influence both extrinsic and intrinsic apoptotic pathways via increased free radicals production. Increased cleaved caspase-8 and 9 protein expression levels after 24 h of treatment with **A1** suggest that this molecule could trigger both intrinsic and extrinsic apoptosis by increasing cleaved caspase-3 (Figure 7). When the gene expression of apoptotic and pro-apoptotic proteins was examined, Blc-2 was found to be down-regulated, while pro-apoptotic caspase-3,8,9,12, and Bax were up-regulated. Bcl-2 protein family controls the apoptosis sensitivity as a regulator of MMP. After disruption of MMP, the mitochondrial permeability transition pores open in order to allow the flow of apoptotic factors^13^^,^^29^. After the treatment, the Bax/Bcl-2 ratio is increased (by an elevated Bax gene expression and reduced Bcl-2 gene expression) and the caspase-3 triggered the signalling cascade as an effector of the apoptosis pathway. (Figure 9).

**Figure 8.**
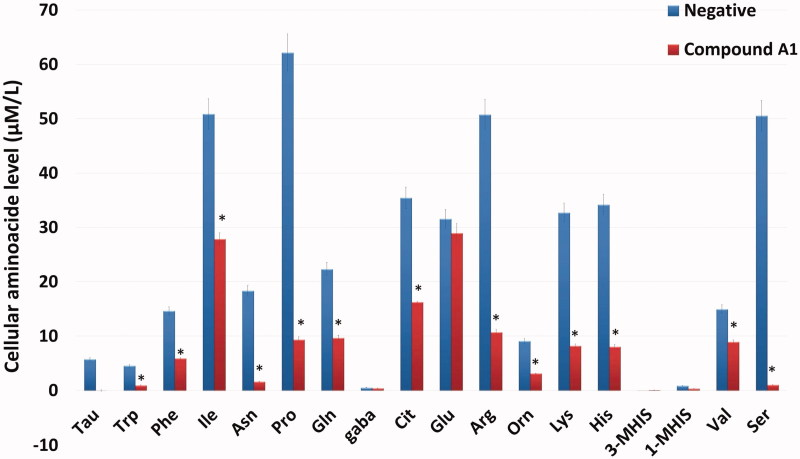
Intracellular free amino acid profiling of HeLa cells: The absolute levels of intracellular free amino acids were quantified using LC-MS/MS. All data are presented as mean ± SD values (*n* = 3). Error bars indicate the SD. *p < .01 compared with the control.

**Figure 9.**
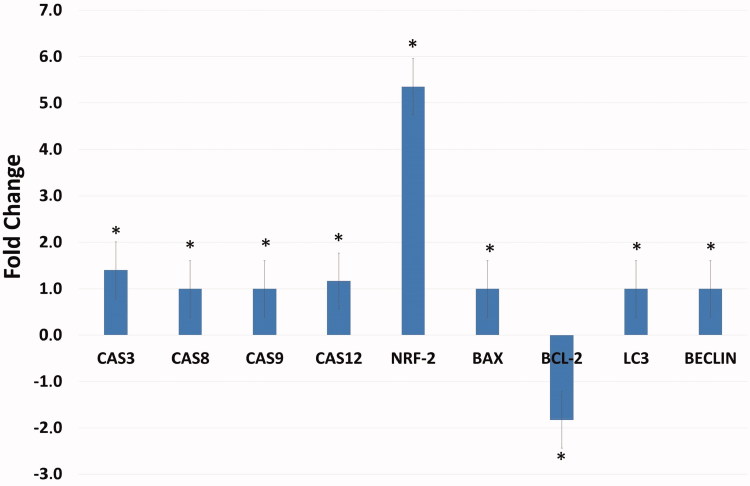
Effects of compound **A1** on the expression of apoptotic (caspase-3,8,9,12, and Bax), anti-apoptotic (Bcl-2) and autophagy (LC3, Beclin) related genes. The data are presented as the mean ± SD. (*n* = 3) *p < .01 compared with the control.

**Figure 10.**
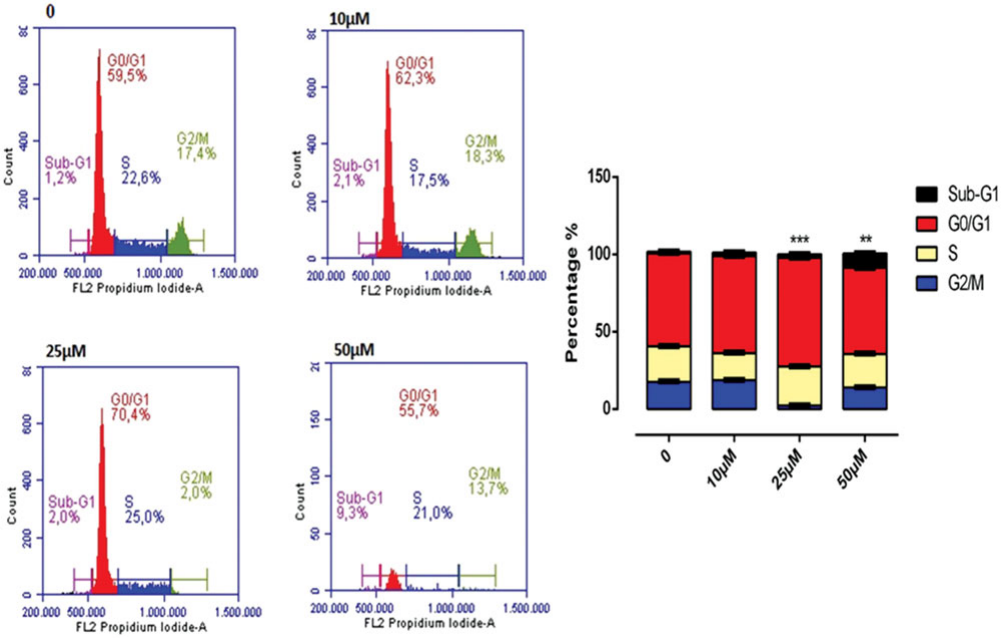
Cell Cycle Analysis of HeLa cells treated with **A1** at 0, 10, 25, and 50 µM concentration treatment after 24 h. All data are expressed as mean ± SD values from three independent experiments. *p*-values of less than .05 (**p < .05, ***p < .01, compared with the control group) are considered significant.

Administration of anti-alkaline agents such as staurosporine to cancer cells induces apoptosis in these cells by disrupting the intracellular homeostasis and metabolism^31^. Amino acids play a significant role in the cellular mechanism since they represent the building blocks of protein synthesis in addition to being intermediary metabolites inducing other bio-synthetic reactions^32^. The intracellular amino acid level is a required signal for regulation of mTOR kinase activity. mTOR kinase activity is also controlled by growth factors^33^^,^^34^. Amino acids suppress autophagy, while absence of amino-acids can stimulate autophagy^35^. When the intracellular amino acid levels were assessed using LC-MS/MS, administration of **A1** was found to be associated with an increase in tyrosine levels and a reduction in other amino acids. This increase in tyrosine may be due to an inhibition of the tyrosine kinase signalling pathway. When the intracellular amino-acid concentration falls, autophagy is activated to produce the amino acids required for cellular survival^36^. Genes coupled with autophagy, i.e. Beclin-1 and LC3, induce autophagy and may suppress the tumour growth. Therefore they are considered to represent a potential therapeutic target in cancer management^37^^,^^38^. In our study all amino acids except tyrosine were reduced, with a subsequent gene expression of Beclin-1 and LC3, indicating that autophagy was taking place.

DNA damage caused by increasing intracellular free radicals leads to p53 expression, ceasing the cell cycle at G1 and apoptosis via PI3K/AKT/mTOR pathway inhibition^7^^,^^25^^,^^39^. The G1 arrest in the cell cycle results in the inhibition of cancer cell proliferation^40^. In this study, the increase in H2AX levels due to administration of **A1** is indicative of DNA damage. Arrest of the cell cycle at G1 is a well-known consequence of DNA damage associated with p53 induction. Again, in the immunofluorescence analysis, HeLa cells treated with Compound **A1** had increasing numbers of H2AX foci as well as increasing cleaved-PARP levels (Figure 6), showing that DNA damage and related apoptosis have occurred.

In conclusion, the results of this study show that the sulfonamide derivative **A1** was able to reduce cancer cell proliferation and induce apoptosis. Based on these results, initial testing in hypoxia models and 3-dimensional cell cultures with subsequent animal experiments involving the use of this molecule may pave the way for novel studies useful in the design of anti-cancer therapeutics.
